# Regulation of NMDA Receptor Plasticity in the BNST Following Adolescent Alcohol Exposure

**DOI:** 10.3389/fncel.2019.00440

**Published:** 2019-10-04

**Authors:** Kathryn L. Carzoli, Nathan M. Sharfman, Mollie R. Lerner, Miriam C. Miller, Eleanor B. Holmgren, Tiffany A. Wills

**Affiliations:** ^1^Department of Cell Biology and Anatomy, LSU Health Sciences Center New Orleans, New Orleans, LA, United States; ^2^Neuroscience Center of Excellence, LSU Health Sciences Center New Orleans, New Orleans, LA, United States

**Keywords:** adolescence, alcohol, BNST, NMDA receptor, sex differences, stress

## Abstract

Persistent alterations in synaptic plasticity and neurotransmission are thought to underlie the heightened risk of adolescent-onset drinkers to develop alcohol use disorders in adulthood. The bed nucleus of the stria terminalis (BNST) is a compelling region to study the consequences of early alcohol, as it is innervated by cortical structures which undergo continued maturation during adolescence and is critically involved in stress and negative affect-associated relapse. In adult mice, chronic ethanol induces long-term changes in GluN2B-containing NMDA receptors (NMDARs) of the BNST. It remains unclear, however, whether the adolescent BNST is susceptible to such persistent alcohol-induced modifications and, if so, whether they are preserved into adulthood. We therefore examined the short- and long-term consequences of adolescent intermittent ethanol exposure (AIE) on NMDAR transmission and plasticity in the BNST of male and female mice. Whole-cell voltage clamp recordings revealed greater glutamatergic tone in the BNST of AIE-treated males and females relative to air-controls. This change, which corresponded to an increase in presynaptic glutamate release, resulted in altered postsynaptic NMDAR metaplasticity and enhanced GluN2B transmission in males but not females. Only AIE-treated males displayed upregulated GluN2B expression (determined by western blot analysis). While these changes did not persist into adulthood under basal conditions, exposing adult males (but not females) to acute restraint stress reinstated AIE-induced alterations in NMDAR metaplasticity and GluN2B function. These data demonstrate that adolescent alcohol exposure specifically modifies NMDARs in the male BNST, that the plastic changes to NMDARs are long-lasting, and that they can be engaged by stress.

## Introduction

The adolescent response to alcohol is unique from adults, both behaviorally and neurally. For instance, adolescents appear less susceptible to certain adverse effects of alcohol (e.g., motor impairments, hangover symptoms, and sedative properties), yet show heightened sensitivity to alcohol’s rewarding effects (Crews et al., 2016). In contrast, adolescents appear more vulnerable than adults to ethanol-induced impairments in learning and memory (Sircar and Sircar, 2005) and neural plasticity (Pyapali et al., 1999). This combination of attenuated sensitivity to cues which moderate alcohol intake and greater sensitivity to disruptions in brain plasticity, culminates in a propensity for adolescents to “binge” drink during a time when aversive neural consequences could be long-lasting. Indeed, repeated binge-like ethanol exposure during adolescence has been linked to disrupted neurotransmission in the medial prefrontal cortex of adults (Trantham-Davidson et al., 2017), as well as to enduring alterations in neuronal morphology and synaptic plasticity in the adult hippocampus (Risher et al., 2015).

Developmental changes which occur across the adolescent brain, in part, contribute to age-related differences in alcohol sensitivity. This is because alcohol acts on many of the same neurotransmitter systems that undergo marked changes during adolescence (Spear, 2013). The effect of alcohol on one type of glutamate receptor in particular, the *N*-methyl-D-aspartate receptor (NMDAR), is critical as NMDARs are involved in regulating synaptic plasticity, remodeling immature synaptic circuits, and, ultimately, for certain forms of learning and memory (Hansen et al., 2017). NMDARs are tetramers that contain two obligatory GluN1 subunits co-assembled with GluN2 (GluN2A–D) and/or GluN3 (GluN3A–B) (Paoletti, 2011). The four GluN2 subunits are major determinants of the functional heterogeneity of NMDARs, and show distinct differences in their regional and developmental expression (Paoletti et al., 2013). An important developmental switch in NMDAR composition—from primarily GluN2B-containing to predominantly GluN2A-containing—alters functional properties of the receptor and reduces synaptic plasticity (Paoletti et al., 2013). Interestingly, while the subunit shift is prevalent throughout much of the brain (including the ethanol-sensitive cortex, hippocampus, and lateral amygdala), other alcohol-responsive brain regions, such as the central nucleus of the amygdala (CeA) and the bed nucleus of the stria terminalis (BNST), appear resistant to this change, retaining significant levels of GluN2B that persist into adulthood (Lopez de Armentia and Sah, 2003; Wills et al., 2013).

The NMDA receptor is a known target of ethanol’s action in the brain, the consequences of which vary depending on the duration of action and age of exposure. Acutely, ethanol inhibits NMDARs and NMDAR-dependent synaptic plasticity across several alcohol-sensitive brain regions (Wills and Winder, 2013). In the BNST, this inhibition is greater in slices from adolescent mice relative to adults (Wills et al., 2013). Conversely, chronic ethanol leads to a compensatory increase in NMDARs, largely through enhanced GluN2B expression and function (Wills and Winder, 2013). For example, adult male mice undergoing withdrawal from chronic intermittent ethanol (CIE) exhibit specific increases in the number of GluN2B subunits within the BNST (Kash et al., 2009), as well as augmented GluN2B-dependent long-term potentiation (LTP) (Wills et al., 2012).

The BNST is uniquely suited for investigating the effects of adolescent alcohol use as it is a sexually dimorphic structure that sits at the intersection of cortical inputs and hypothalamic targets (Ch’ng et al., 2018). As such, transmission in the BNST critically regulates negative affect and stress responsiveness—two factors central to the progression and development of alcohol use disorders (AUDs) as both are primary contributors to relapse and, thereby, continued alcohol use (Koob, 2009). Determining how ethanol modulates plasticity in this region during adolescence, and exploring the later consequences of this exposure during adulthood, could provide useful clues for understanding sex differences in the vulnerability to alcohol dependence and relapse. We therefore combined pharmacology with electrophysiology to investigate the effects of ethanol exposure on NMDAR-mediated excitatory transmission within the adolescent BNST of both male and female mice. We then assessed the consequences of this early exposure on adult BNST plasticity under basal conditions, as well as following known triggers of relapse in humans (e.g., alcohol re-exposure and stress).

## Materials and Methods

### Animals

Three-week-old (postnatal day [P]21) C57BL/6J mice were obtained from Jackson Laboratories (Bar Harbor, ME, United States); males and females were housed separately, in groups of four, with food and water available *ad libitum*. All procedures were approved by the Animal Care and Use Committee at Louisiana State University Health Sciences Center.

### Adolescent Chronic Intermittent Ethanol Vapor Exposure

Adolescent mice were given a daily injection of either pyrazole + saline (Air-control group, 1 mmol/kg) or pyrazole + ethanol (AIE group, 1 mmol/kg + 0.8 g/kg, respectively) to impair the metabolism of ethanol. Mice were placed back in their home cages where they were left undisturbed for 30 min. Home cages were then placed into chambers filled with either volatilized water or ethanol (20.3 ± 0.2 mg/L); airflow through the chambers was maintained at 5.5 L/min and volatilization was maintained at 1.5 L/min. After 16 h of continuous exposure, mice were removed from the chambers and returned to standard animal housing. Ethanol chamber exposure occurred from 1600–0800 the following day, which allowed for the reliable obtainment of blood ethanol concentrations in the range of 150–185 mg/dL. This protocol was run for two, 4-day cycles of 16-h in-chamber sessions and 8-h out-of-chamber sessions. The two sessions were separated by 3 days of no vapor exposure. Male and female mice were exposed in separate vapor chambers. For adult ethanol re-exposure, mice (P70–75) underwent a single alcohol vapor chamber session (16 h). Brain slices for all experiments were collected during acute withdrawal (4–5 h following removal from the vapor chambers) unless otherwise noted.

### Adult Acute Restraint Stress

To evaluate the effects of stress on animals with a history of adolescent intermittent ethanol (AIE) exposure, adult mice (P70–75) previously subjected to either air (Air-Str) or ethanol (AIE-Str) were restrained in well-ventilated 50-ml conical tubes and left undisturbed for 1 h. After the restraint period, mice were transferred to their cages for 1 h before slicing for electrophysiology.

### Electrophysiology

#### Slice Preparation

Mice were transported from the animal colony to the laboratory and placed in a sound-attenuated cubicle for 1 h. Mice were then decapitated under isoflurane, and their brains were quickly removed and placed in ice-cold oxygenated (5% Co2/95% O2 mix) slicing solution containing (in mM) 20 NaCl, 0.46 KCl, 26 NaHCO3, 1.34 NaH2PO4, 1 MgCl26H2O, 183 sucrose, and 10 glucose. Coronal slices (300 μm in thickness) containing the dorsolateral BNST (dlBNST; bregma, 0.26–0.02 mm) were obtained using a vibrating microslicer (Leica Biosystems, Bannockburn, IL, United States). After sectioning, slices were transferred to a heated holding chamber (∼29°C) containing oxygenated artificial cerebral spinal fluid (ASCF) for 1 h prior to both whole-cell and field recordings. For recordings, slices were transferred to an interface recording chamber where they were perfused with heated (∼36°C), oxygenated ACSF containing (in mM) 124 NaCl, 4.1 KCl, 26 NaHCO3, 1 NaH2PO4, 2.6 CaCl2, 2.4 MgSO4, and 25 glucose; pH 7.2–7.4, 290–310 mOsm at a rate of ∼2 ml/min. Membrane current and voltage were sampled at 10 kHz and low-pass filtered at 2 kHz.

#### Whole Cell Recordings

Recording electrodes (5–7 MΩ; Flaming-Brown Micropipette Puller, Sutter Instruments) were filled with Cs + gluconate internal solution containing (in mM) 117 Cs + gluconate, 20 HEPES, 0.4 EGTA, 5 TEA, 2 MgCl2, 4 NaATP, 0.3 NaGTP; pH 7.25–7.35, 285–290 mOsm. Spontaneous excitatory postsynaptic currents (sEPSCs) were recorded at −70 mV in the presence of picrotoxin (25 μM). Miniature excitatory postsynaptic currents (mEPSCs) were recorded at −70 mV in the presence of picrotoxin (25 μM) and tetrodotoxin (500 nM). Spontaneous and miniature EPSCs were selected using an event detection template, the average frequency and amplitude of which were calculated and averaged across five 2-min periods. NMDAR-mediated excitatory postsynaptic currents (NMDAR-EPSCs) were evoked by local fiber stimulation with a bipolar nichrome electrode (5–30 pA with a 100–150 μs duration, 0.0167 Hz) at a holding potential of +40 mV in normal ACSF containing picrotoxin (25 μM) and NBQX (10 μM); an electrical stimulation intensity that elicited 100–300 pA event amplitudes was used. NMDAR-EPSCs were analyzed by measuring the peak amplitude of the synaptic response, and then normalizing that value to the baseline period. The inhibition of NMDAR currents by Ro 25–6981 (2 μM) was determined by calculating the percent change in EPSC amplitude from 5 min before Ro addition to 5 min of the peak drug effect (last 5 min of washout). This analysis point was chosen as it has been shown to be the maximal point of drug effectiveness in the BNST (Kash et al., 2008; Wills et al., 2013); antagonism by Ro 25–6981 is permanent and the drug does not washout after application (Karakas et al., 2011; Zhu et al., 2016). GluN2B-EPSCs were obtained by subtracting NMDAR-EPSCs in the presence of Ro 25–6981 from those in its absence. The GluN2B/NMDAR ratio was then determined by dividing the peak GluN2B-EPSC amplitude by the peak NMDAR-EPSC amplitude. For temporal summation experiments, evoked NMDAR-EPSCs were recorded in response to bursts of stimulation (10 pulses) at 10, 20, and 40 Hz. Peak EPSC amplitudes were normalized to the first pulse in each stimulation burst. Area under the curve values were calculated and compiled to generate a summation index for each frequency. Series resistance was monitored over the duration of all whole-cell recordings, and collected data were not included if changes in this value were greater than 20%.

#### Field Potential Recordings

A bipolar stainless-steel stimulating electrode and a borosilicate glass recording electrode (1–2 MΩ) filled with ACSF were placed in the dlBNST to elicit and record an extracellular field response, respectively. For LTP experiments, baseline responses to a stimulus intensity (duration, 50 μsec) that produced ∼40% of the maximum response were recorded for at least 20 min at a rate of 0.05 Hz. After acquisition of a stable baseline, LTP was induced by a 1-sec tetanus of two 100-Hz trains, with a 20-s inter-train interval at the same intensity used for baseline test pulses. Analyses were then conducted to determine the percent change in field excitatory postsynaptic potentials, from baseline to 0–10 min and 51–60 min post-tetanus. Experiments in which the fiber volley (N1) changed by ≥20% were discarded.

### Western Blotting

For protein extraction, 0.33 mm tissue punches (slice thickness, 500 μm) obtained from the dlBNST 4–5 h following the final vapor chamber session were homogenized in homogenization buffer. Proteins were resolved by SDS-PAGE (10%) and transferred to nitrocellulose membranes, which were blocked in 5% milk in TBST and incubated with the appropriate primary and secondary antibodies; infrared-conjugated secondary antibodies (LiCor Biosciences, Lincoln, NE, United States) were used for detection with the Odyssey system (LiCor Biosciences). Densitometry was performed using Image J (National Institutes of Health, Bethesda, MD, United States) on images linearly adjusted for brightness and contrast. To combine blocks of experiments across blots, signals were normalized to GAPDH (1:10 000; Abcam, Cambridge, MA, United States) and calculated as a percentage of the control values on their respective blot. The following primary antibodies were used: GluN1 (1:2000; BD, Franklin Lakes, NJ, United States), GluN2B (1:2000; BD), and GluN2A (1:2000; Millipore Sigma, Burlington, MA, United States) (Wills et al., 2012).

### Statistical Analysis

Clampfit 9.0 (Axon Instruments) was used for the analysis of electrophysiological data. All values are presented as means ± SEM, and a *p*-value < 0.05 was considered significant. Normality and the equality of variances were assessed, and statistical tests (including mostly unpaired *t*-tests, with Welch’s correction for unequal variance, and one- or two-way ANOVAs) were chosen accordingly. The n for electrophysiological recordings is a reflection of the number of slices or cells used per group. All experimental groups contained at least four mice.

## Results

### AIE Alters Glutamatergic Tone in the dlBNST of Male and Female Mice

**FIGURE 1:**
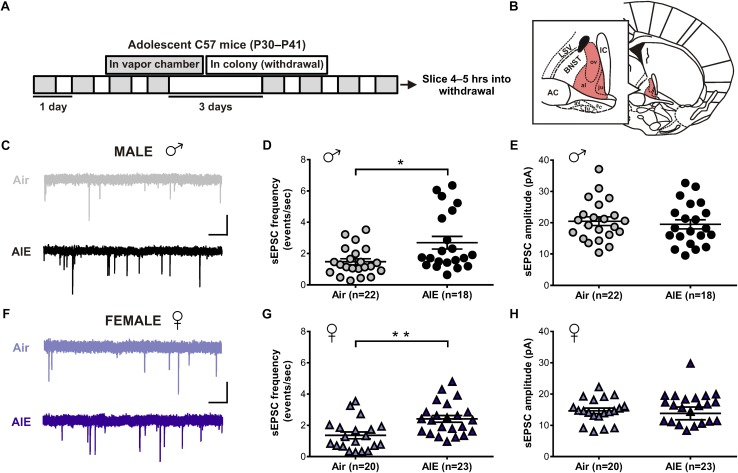
Spontaneous EPSCs in the dorsolateral BNST (dlBNST) following acute withdrawal from adolescent chronic intermittent ethanol treatment (AIE) in the male and female mice. **(A)** Schedule of ethanol and water vapor exposure; mice were placed in a chamber filled with volatilized ethanol (AIE) or volatilized water (Air) for four 16-h blocks of exposure, separated by four 8-h periods of withdrawal. **(B)** Illustration of a coronal brain section with the dlBNST shown in red. AC, anterior commissure; IC, Internal capsule; LSV, lateral septal nucleus; note, lowercase abbreviations refer to subdivisions of the BNST. Representative sEPSC traces from control (top, gray) and intermittent ethanol-exposed (bottom, black) male **(C)** and female **(F)** mice; vertical scale bar = 15 pA, horizontal scale bar = 500 ms. Quantification of sEPSC frequency **(D,G)** and amplitude **(E,H)** in dlBNST neurons of male (circles) and female (triangles) mice. Lines show mean (±SEM), symbols indicate significant differences determined by unpaired *t*-tests; ^∗^*p* < 0.05, ^∗∗^*p* < 0.01.

Given the BNST’s role in alcohol abuse, along with numerous studies showing an effect of alcohol on glutamatergic transmission, we first sought to determine whether acute withdrawal from AIE altered basal glutamate release in the BNST. In order to do so, adolescent male and female mice were exposed to chronic intermittent ethanol (AIE) or air (Figure 1A). Five hours after the final vapor chamber session, whole-cell voltage-clamp recordings were obtained from neurons within the dlBNST (Figure 1B) to assess sEPSCs. Using this approach, we found a significant increase in the frequency (Welch’s corrected *t*-test: *t*[28.6] = 2.724; *p* = 0.011, Figures 1C,D), but not amplitude (*t*[41] = 0.493; *p* = 0.6249, Figures 1C,E), of sEPSCs in AIE-exposed male mice (8 mice, *n* = 22) when compared to male air-controls (8 mice, *n* = 18). As these data illustrated that the effect of AIE on sEPSC frequency is driven by a subpopulation of cells, we next evaluated whether specific cell properties (capacitance, membrane resistance, and holding current) were correlated with this change. No correlation was observed between sEPSC frequency and membrane resistance or holding current. Moreover, while a significant positive correlation was observed between capacitance and sEPSC frequency for both air controls (*r*^2^ = 0.211, *F*[1,20] = 5.35, *p* = 0.032) and AIE-treated mice (*r*^2^ = 0.24, *F*[1,18] = 5.63, *p* = 0.029), there was no differences between groups in slope (*F*[1,38] = 1.783, *p* = 0.19). Thus, capacitance alone cannot uniquely identify the population of high sEPSC frequency cells in AIE treated male mice. Additionally, we found a trend for an increase in the frequency (Welch’s corrected *t*-test: *t*[18.1] = 1.894; *p* = 0.0743, data not shown), but not amplitude (*t*[31] = 0.0313, *p* = 0.975, data not shown), of miniature excitatory postsynaptic currents (mEPSCs) when AIE-exposed male mice (8 mice, *n* = 15) were compared to male air-controls (7 mice, *n* = 18). Similarly, female mice exposed to AIE (11 mice, *n* = 23) displayed a significant increase in the frequency (*t*[41] = 3.424, *p* = 0.001, Figures 1F,G), but not amplitude (*t*[41] = 0.36; *p* = 0.721, Figures 1F,H), of sEPSCs relative to female air-controls (8 mice, *n* = 20). These findings suggest that acute withdrawal from AIE alters glutamate release in the dlBNST of both male and female mice.

### AIE Enhances GluN2B-NMDARs in the dlBNST of Male Mice

**FIGURE 2:**
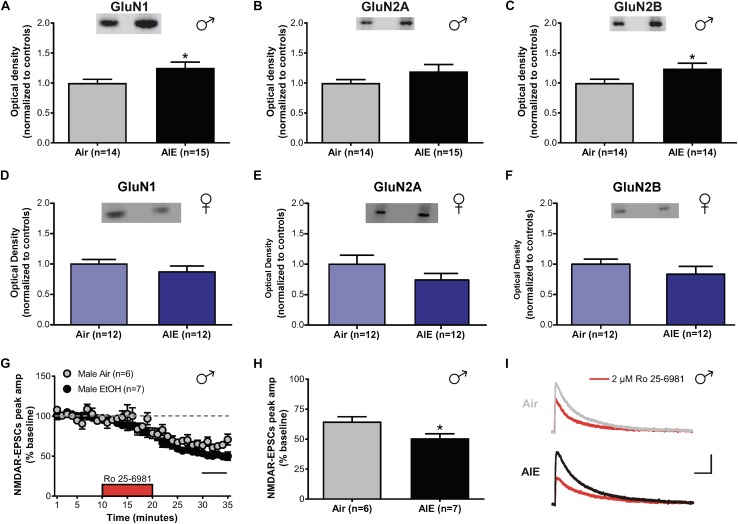
NMDAR subunit expression and transmission in the dlBNST of male and female mice following acute withdrawal from AIE. Western blot analysis of GluN1 **(A)**, GluN2A **(B)**, GluN2B **(C)** subunit expression from air- and AIE-treated male and female mice **(D–F)**. Tissue was collected 5 h following the final vapor chamber exposure, during acute withdrawal. Mean (± SEM) optical density values for protein bands are shown as percent of the corresponding controls run in the same blot. Ro 25–6981 (2 μM) was applied to dlBNST slices from adolescent mice exposed to chronic intermittent ethanol (AIE) or volatilized water (Air); evoked NMDAR-isolated EPSCs were then recorded. **(G)** Time course of NMDAR-EPSC peak amplitudes in Air- and AIE-treated groups during a 10-min application of Ro 25–6981. Data points are the mean ± SEM. **(H)** Inhibition of NMDAR currents by Ro 25–6981, determined by calculating the percentage change in EPSC amplitude from 5 min before Ro addition to 5 min of the peak drug effect (last 5 min of washout). **(I)** Representative traces of NMDAR-EPSCs recorded from mice exposed to Air (top) and AIE (bottom) before (gray and black traces, respectively) and after (red traces) application of Ro 25–6981; vertical scale bar = 100 pA, horizontal scale bar = 200 ms. Symbols indicate significant differences determined by unpaired *t*-tests; ^∗^*p* < 0.05.

In the adult BNST, sensitivities of glutamatergic transmission to the effects of both acute and chronic ethanol are GluN2B-dependent (Wills et al., 2012, 2013). As different GluN2 subunits have distinct signaling pathways and decay kinetics, determining whether similar changes occur in adolescents undergoing acute withdrawal from AIE is critical to understand how ethanol modulates activity in the developing brain. To investigate this, we tested for differences in the expression levels of GluN1, GluN2A, and GluN2B between air- and AIE-exposed male and female mice in the dlBNST using western blot analysis (Supplementary Figure S1). We found a significant increase in GluN1 (*t*[27] = 2.207; *p* = 0.036, Figure 2A) and GluN2B (*t*[26] = 2.189; *p* = 0.038, Figure 2C) subunits in the dlBNST of male mice following AIE (GluN1 [*n* = 15 mice]: 1.252 ± 0.095 and GluN2B [*n* = 14]: 1.24 ± 0.090) compared to air-controls (GluN1 [*n* = 14 mice]: 1 ± 0.060 and GluN2B [*n* = 14]: 1 ± 0.062), but no change in GluN2A protein expression (AIE [*n* = 15 mice]: 1.192 ± 0.115 versus Air [*n* = 14 mice]: 1 ± 0.055; *t*[27] = 1.471; *p* = 0.153, Figure 2B). In contrast, female mice showed no significant difference in GluN1 (*t*[22] = 1.059; *p* = 0.301, Figure 2D), GluN2A (*t*[22] = 1.423; *p* = 0.169, Figure 2E), or GluN2B (*t*[22] = 1.09; *p* = 0.288, Figure 2F) subunits in the BNST following AIE (GluN1 [*n* = 12 mice]: 0.871 ± 0.096, GluN2A [*n* = 12 mice]: 0.742 ± 0.107, and GluN2B [*n* = 12 mice]: 0.957 ± 0.153) compared to air-controls (GluN1 [*n* = 12 mice]: 1 ± 0.075, GluN2A [*n* = 12 mice]: 1 ± 0.147, and GluN2B [*n* = 12 mice]: 1 ± 0.082). These data demonstrate a sex-specific enhancement of GluN2B and GluN1 subunits following AIE in male mice.

To determine if the observed increase in GluN2B/GluN1 altered NMDAR transmission, whole-cell recordings of isolated evoked NMDAR-EPSCs in the male adolescent dlBNST were obtained from air-control and AIE male mice. Baseline evoked NMDAR-EPSCs were significantly larger in AIE-treated mice (6 mice, *n* = 7, 480 ± 52.6 pA) compared to air-controls (5 mice; *n* = 6, 210 ± 36.8 pA; *t*[11] = 4.061, *p* = 0.002). Following 10 min of stable baseline, Ro 25–6981 (2 μM, GluN2B antagonist) was bath applied to investigate the presence of functional GluN2B subunits. Using this approach, we observed a significant difference in the Ro 25–6981-mediated inhibition of NMDAR-EPSC amplitudes in AIE male mice compared to those that received only air (51 ± 4% of baseline peak in AIE-treated versus 65 ± 4% of baseline peak in air-controls, *t*[11] = 2.613; *p* = 0.024, Figures 2G–I). We found no significant difference in the decay time of NMDAR-EPSCs in dlBNST neurons from AIE-exposed mice when compared to air-controls (AIE weighted tau, 282 ± 30 ms versus Air weighted tau, 244 ± 23 ms; *t*[11] = 0.968, *p* = 0.354, data not shown) suggesting that the greater GluN2B-NMDAR inhibition observed in AIE mice was due to an increase in heterotrimeric GluN1/GluN2A/GluN2B-NMDARs as it has been previously postulated that changes in this type of receptor have no effect on decay kinetics (Kash et al., 2008). Further, the ability of Ro 25–6981 to alter EPSC decay kinetics did not significantly differ between groups (% change in NMDAR-EPSC weighted tau compared to baseline: AIE, −9 ± 5% versus Air, −10 ± 5%; *t*[11] = 0.158, *p* = 0.878). The fact that Ro 25–6981 altered NMDAR-EPSC decay kinetics in both AIE and Air groups pointing to an underlying population of heterodimeric GluN1/GluN2B-NMDARs. Together, the heightened GluN2B inhibition and enhanced GluN2B/GluN1 subunit expression suggest a functional, GluN2B-NMDAR-specific increase following AIE in male mice. These findings are similar to those observed in the ventral BNST of adult mice undergoing acute withdrawal from chronic ethanol (Kash et al., 2009).

### AIE Enhances NMDAR-Mediated Metaplasticity in the dlBNST of Male Mice

**FIGURE 3:**
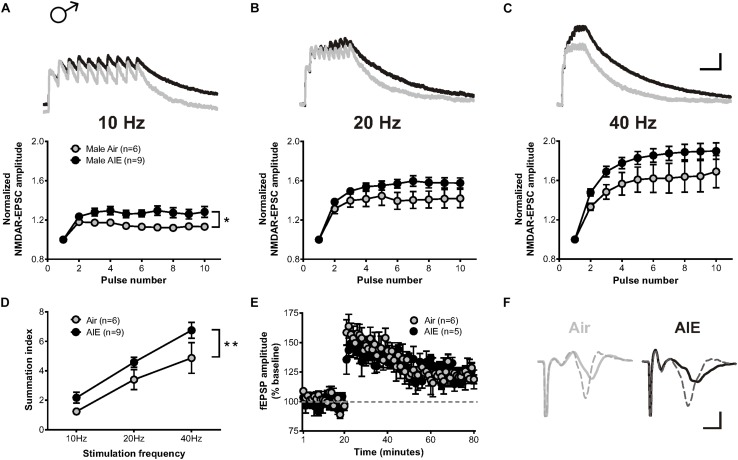
Temporal summation of NMDAR-EPSCs and LTP following acute withdrawal from AIE in the male dlBNST. **(A–C)** Representative traces (top; Air gray, AIE black) and pooled data (bottom) demonstrating the ability of 10 stimulation pulses given at 10, 20, and 40 Hz to enhance the temporal summation of NMDAR-EPSCs during withdrawal from AIE; vertical scale bar = 200 pA, horizontal scale bar = 200 ms. Symbols indicate significant differences determined by a two-way repeated measures ANOVA; ^∗^*p* < 0.05. **(D)** Plot of the summation index across different stimulation frequencies and treatments demonstrating a significant increase in temporal summation following AIE. A two-way ANOVA revealed a significant effect of treatment; ^∗∗^*p* < 0.01. **(E)** Time course showing no difference in synaptic field potentials at baseline (20 min) and after the induction of LTP by high-frequency stimulation (two 1-s trains at 100 Hz) in the dlBNST of mice from Air- and AIE groups. **(F)** Representative traces recorded from Air (gray) and AIE (black) mice before (solid) and 60 min after (dashed) the high frequency tetanus; vertical scale bar = 0.3 mV, horizontal scale bar = 2 ms. All data points represent the mean ± SEM.

A previous study described that exposure to chronic intermittent, but not continuous, ethanol increases the temporal summation of NMDAR-mediated EPSCs in the adult ventral BNST (Kash et al., 2009). As the temporal summation of NMDARs has been proposed as a marker of metaplasticity (Philpot et al., 2007) and monitor both synaptic and extrasynaptic NMDAR populations, we asked whether acute withdrawal from AIE would induce similar changes in the dlBNST. To answer this question, bursts of stimulation at 10, 20, and 40 Hz were delivered to cells voltage clamped at +40 mV in order to evoke NMDAR-EPSCs. Two-way repeated measure ANOVAs on recordings from AIE-exposed mice (5 mice, *n* = 9) revealed a significant increase in the temporal summation of NMDAR-EPSCs at 10 Hz (*F*[1,13] = 6.332, *p* = 0.026) and a trend toward an increase at 20 and 40 Hz (F[1,13] = 2.908, *p* = 0.1119 and *F*[1,13] = 2.98, *p* = 0.108, respectively) when compared to corresponding air-controls (4 mice, *n* = 6; Figures 3A–C). Further investigation of the charge transfer elicited in response to these stimulation frequencies (measured as the area under the normalized temporal summation curve of each frequency [i.e., summation index (Kash et al., 2009)]; Figure 3D) uncovered a significant effect of frequency (*F*[2,39] = 27.49, *p* < 0.001) and a significant difference between groups (*F*[1,39] = 8.655, *p* = 0.006), signifying potential sensitization of NMDAR-dependent metaplasticity during acute withdrawal from AIE. Baseline NMDAR-mediated EPSCs were not significantly different between AIE (410 ± 72.46 pA) and control male mice (370 ± 67.87 pA; *t*[10] = 0.242, *p* = 0.739).

LTP, a form of NMDAR-dependent plasticity at synapses throughout the brain, is increased in the dlBNST following chronic intermittent ethanol exposure in adults (Wills et al., 2012). We investigated whether AIE would yield similar results by stimulating local afferents with a moderate intensity tetanus protocol (two 100-Hz, 1-s trains separated by a 20 s inter-train interval). In contrast to adults, AIE (5 mice, *n* = 5) exerted no influence over either the ability or degree of LTP induction relative to air-controls (4 mice, *n* = 6) (0–10 min post-tetanus: [*t*(9) = 0.6997, *p* = 0.502], 51–60 min post-tetanus: [*t*(9) = 0.754, *p* = 0.47]; Figures 3E,F). This finding was unexpected given the AIE-induced enhancement in GluN2B-transmission and temporal summation. However, the population activity observed in the field recordings of our LTP experiment may not necessarily mirror activity obtained at the individual cell level (measured using whole-cell electrophysiology). Moreover, the mechanisms responsible for mediating metaplasticity and synaptic plasticity are likely divergent (Philpot et al., 2007; Abela and Chudasama, 2014) and involve different contributions of synaptic versus extrasynaptic NMDARs.

### NMDAR-Mediated Metaplasticity in the dlBNST of AIE Mice Is Sex-Specific

**FIGURE 4:**
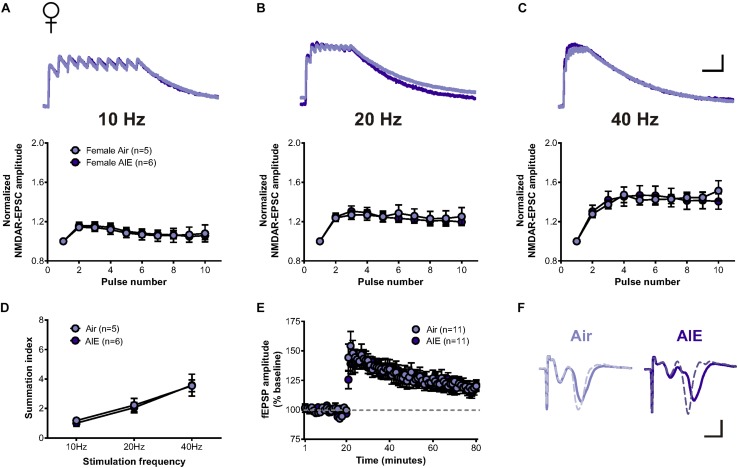
Temporal summation of NMDAR-EPSCs and LTP following acute withdrawal from AIE in the female dlBNST. **(A–C)** Representative traces (top; Air light purple, AIE dark purple) and pooled data (bottom) demonstrating the lack of difference between female groups in response to 10 stimulation pulses given at 10, 20, and 40 Hz to induce temporal summation of NMDAR-EPSCs; vertical scale bar = 200 pA, horizontal scale bar = 200 ms. **(D)** Plot of the summation index across stimulation frequencies and treatments demonstrating no significant difference in temporal summation following AIE in females. **(E)** Time course showing no difference in synaptic field potentials at baseline (20 min) and after the induction of LTP by high-frequency stimulation (two 1-s trains at 100 Hz) in the dlBNST of mice from Air- and AIE groups. **(F)** Representative traces recorded from Air (light purple) and AIE (dark purple) female mice before (solid) and 60 min after (dashed) the high frequency tetanus; vertical scale bar = 0.3 mV, horizontal scale bar = 2 ms. All data points represent the mean ± SEM.

Since the BNST is a highly sexually dimorphic brain region (Allen and Gorski, 1990; Hines et al., 1992; Lebow and Chen, 2016), we next asked whether sex conferred differential sensitivity of AIE-induced plasticity in the BNST. In response to the same stimulation bursts delivered to cells following AIE in males, two-way repeated measure ANOVAs determined no significant increase in the temporal summation of NMDAR-EPSCs evoked in dlBNST neurons following AIE in female mice (5 mice, *n* = 6) when compared to corresponding air-controls (4 mice, *n* = 5) (10 Hz: *F*[1,9] = 0.012, *p* = 0.914; 20 Hz: *F*[1,9] = 0.034, *p* = 0.857; and 40 Hz: *F*[1,9] = 0.0001, *p* = 0.991, Figures 4A–C). Further investigation of charge transfer (Figure 4D) exposed a significant effect of frequency (*F*[2,27] = 15.45, *p* < 0.001) but no significant difference between groups (*F*[1,27] = 0.073, *p* = 0.79). Baseline NMDAR-mediated EPSCs were not significantly different between AIE (302 ± 60.42 pA) and control female mice (388 ± 88.6 pA; *t*[9] = 0.825, *p* = 0.431), nor between male and female mice (two-way ANOVA: sex *F*[1,19] = 0.331, *p* = 0.43; AIE *F*[1,19] = 0.091, *p* = 0.766).

Similar to our investigation of the impact of AIE on LTP within the male dlBNST, we found no significant influence of AIE in female mice (8 mice, *n* = 11) when compared to air-controls (7 mice, *n* = 11) (0–10 min post-tetanus: [*t*(20) = 0.732, *p* = 0.473], 51–60 min post-tetanus: [*t*(20) = 0.234, *p* = 0.817], Figures 4E,F). These findings provide further support to the sexually dimorphic nature of the BNST and demonstrate that functional disparities in ethanol-sensitive pathways arise early, during critical periods of development.

### Persistence of AIE-Induced Changes in NMDARs and Metaplasticity in the Adult BNST

**FIGURE 5:**
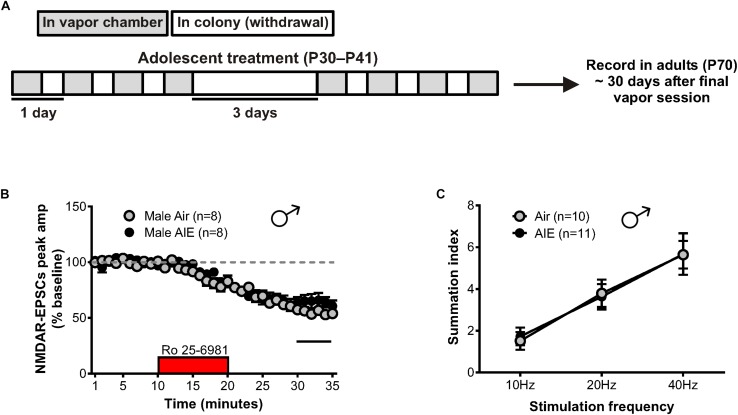
GluN2B transmission and NMDAR metaplasticity in adult male mice with a history of AIE. **(A)** Schedule showing the time course used to evaluate the duration of the plastic changes observed in the male adolescent BNST following chronic intermittent ethanol. Recordings were obtained 30 days after the final vapor chamber session (∼P70). **(B)** Time course of NMDAR-EPSC peak amplitudes in Air- and AIE-treated male adults following a 10-min application of Ro 25–6981. In contrast to the enhancement of GluN2B-mediated transmission observed in adolescent males exposed to chronic intermittent ethanol, there was a trend toward a significant reduction of GluN2B in adults with a history of AIE (unpaired *t*-test comparing group differences of the percentage change in EPSC amplitude from 5 min before Ro addition to the last 5 min of washout [black line]). **(C)** Plot of the summation index across stimulation frequencies and treatments demonstrating no significant difference in adult males with a history of AIE relative to controls. All data points indicate mean ± SEM.

The maintenance of AIE-induced alterations in NMDAR-mediated transmission could contribute to the increased susceptibility of adolescents with a history of alcohol use to develop AUDs (Grant and Dawson, 1997). Thus, we evaluated the duration of the plastic changes observed in the male BNST following AIE by allowing mice to age into adulthood (P70). This timepoint corresponded to 30 days after the last ethanol vapor session (Figure 5A), marking a prolonged period of alcohol abstinence. Whole-cell recordings were then obtained from neurons in the dlBNST, and NMDAR-EPSCs were generated as previously described. Bath application of Ro 25–6981 resulted in no significant difference in the NMDAR-EPSC amplitudes recorded from adult male mice with a history of AIE compared to air-controls (65 ± 5% of baseline peak in AIE-adults [5 mice, *n* = 8] versus 55 ± 3% of baseline peak in air-controls [4 mice, *n* = 8], *t*[14] = 1.641; *p* = 0.123, Figure 5B). Although not significant, a trend for these results was in the opposite direction of that observed in adolescent mice following AIE. Analysis of the decay time of NMDAR-EPSCs recorded from adult male mice with an AIE history revealed no difference when compared to air-control animals (AIE weighted tau, 253 ± 28 ms versus Air weighted tau, 323 ± 34 ms; *t*[14] = 1.579, p = 0.137, data not shown). Additionally, the ability of Ro 25–6981 to alter EPSC decay kinetics did not significantly differ between groups (% change in NMDAR-EPSC weighted tau compared to baseline: AIE, −22 ± 7% versus Air, −18 ± 4%; *t*[14] = 0.437, *p* = 0.669, data not shown). In contrast to our findings of enhanced NMDAR-dependent metaplasticity following chronic intermittent alcohol in adolescents, male adults with an AIE history (*n* = 11) exhibited no difference in temporal summation when compared to air-controls (*n* = 10) (*F*[1,57] = 0.003, *p* = 0.959, Figure 5C).

### Reinstatement of AIE-Induced Metaplasticity Following a Stress Challenge in Adulthood

**FIGURE 6:**
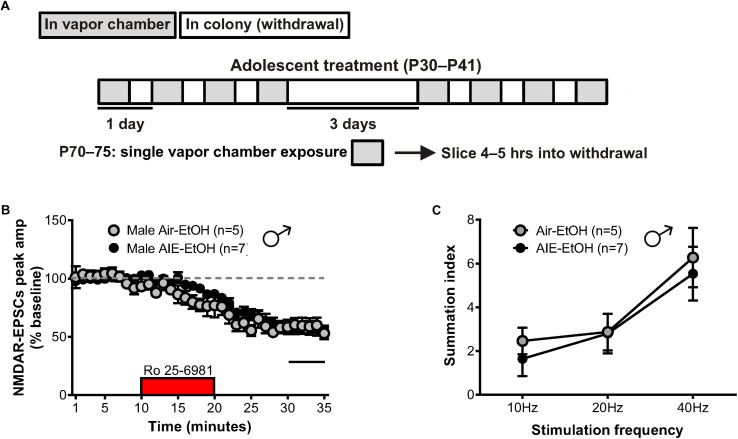
GluN2B transmission and NMDAR metaplasticity in adult male mice with a history of AIE following an ethanol challenge. **(A)** Protocol used to evaluate whether an ethanol challenge (single 16-h vapor chamber session, EtOH) given during a prolonged period of abstinence would re-engage NMDAR plasticity induced by AIE (AIE-EtOH) relative to air controls (Air-EtOH). **(B)** Time course of NMDAR-EPSC peak amplitudes following a 10-min application of Ro 25–6981. **(C)** Plot of the summation index across stimulation frequencies and treatments demonstrating that an alcohol re-exposure had no effect on the temporal summation of NMDA-EPSCs in adult males with a history of AIE. All data points indicate mean ± SEM.

**FIGURE 7:**
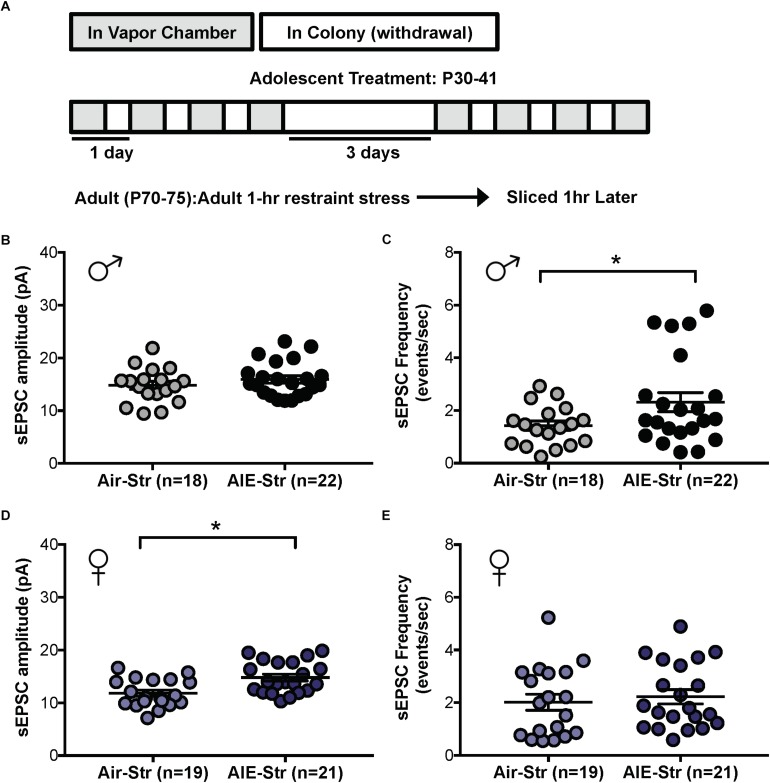
Spontaneous EPSCs in adult male and female mice with a history of AIE following a restraint stress challenge. **(A)** Protocol used to evaluate whether acute restraint stress (1 h, Str) during protracted withdrawal would alter NMDARs in adults with a history of AIE (AIE-Str) relative to air controls (Air-Str). Slices were obtained from mice 1 h after termination of the restraint stress. Quantification of sEPSC frequency **(C)** and amplitude **(B)** in dlBNST neurons of AIE-Str and Air-Str male mice. Quantification of sEPSC frequency **(E)** and amplitude **(D)** in dlBNST neurons of AIE-Str and Air-Str male mice. Lines show mean (± SEM), symbols indicate significant differences determined by unpaired *t*-tests; ^∗^*p* < 0.05.

**FIGURE 8:**
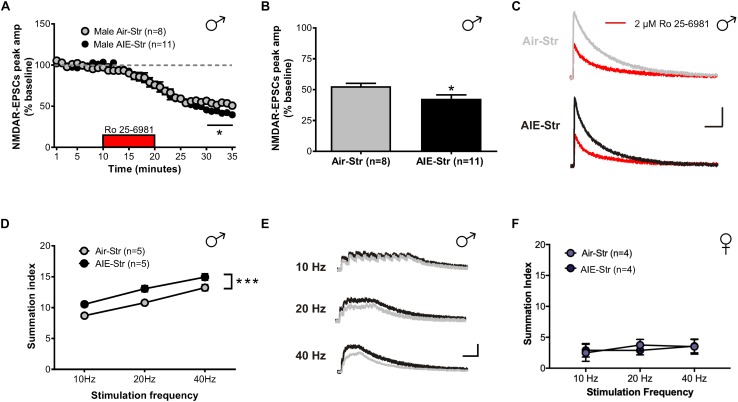
GluN2B transmission and NMDAR plasticity in adult mice with a history of AIE following a restraint stress challenge. **(A)** Time course of NMDAR-EPSC peak amplitudes following a 10-min application of Ro 25–6981. **(B)** Quantification of the inhibition of NMDAR currents produced by Ro 25–6981, determined by calculating the percentage change in EPSC amplitude from 5 min before Ro addition to 5 min of the peak drug effect (last 5 min of washout). **(C)** Representative traces of NMDAR-EPSCs recorded from Air-Str (top) and AIE-Str (bottom) mice before (gray and black traces, respectively) and after (red traces) application of Ro 25–6981; vertical scale bar = 100 pA, horizontal scale bar = 200 ms. **(D)** Plot of the summation index across stimulation frequencies and treatments demonstrating that stress during adulthood was sufficient to restore the sensitization of NMDAR-EPSCs in adults with a history of AIE. A significant effect of treatment was determined by a two-way ANOVA, ^∗∗∗^*p* < 0.001. **(E)** Representative traces (Air-Str gray, AIE-Str black) demonstrating the ability of acute stress to enhance the temporal summation of NMDAR-EPSCs in adult males with a history of AIE; vertical scale bar = 200 pA, horizontal scale bar = 200 ms. **(F)** Plot of the summation index across stimulation frequencies find no significant effect of stress in adult female mice with and without a history of AIE.

Relapse following alcohol abstinence can be triggered by a number of events, including re-exposure to alcohol and stress (Becker, 2008). To examine whether such challenges could re-engage the circuitry activated by AIE, male adults with an AIE history were exposed to a single day of ethanol vapor, 30 days after their last vapor session (Figure 6A). Whole-cell recordings obtained 5–6 h later revealed no change in GluN2B transmission (57 ± 5% of baseline peak in AIE-EtOH adults [4 mice, *n* = 7] versus 58 ± 6% in Air-EtOH controls [4 mice, *n* = 5], *t*[10] = 0.184; *p* = 0.857, Figure 6B) and no difference in temporal summation (*F*[1,30] = 0.422, *p* = 0.521; AIE-EtOH [*n* = 7] versus Air-EtOH [*n* = 5], Figure 6C). On the other hand, male mice with a history of AIE that underwent 1 h of restraint stress (AIE-Str; 10 mice, *n* = 22, Figure 7A) displayed a significant increase in the frequency (Welch’s corrected *t*-test: *t*[30.3] = 2.22; *p* = 0.034, Figure 7B), but not amplitude (*t*[38] = 1.071; *p* = 0.291, Figure 7C), of sEPSCs compared to stressed air-controls (Air-Str; 8 mice, *n* = 18). AIE-stress male mice also exhibited greater GluN2B-mediated transmission (AIE: baseline 309.5 ± 56.8 pA, 8 mice, *n* = 11; Air: baseline 457.6 ± 83.32, 6 mice, *n* = 8) and metaplasticity, showing a significant increase in the Ro 25–6981-induced inhibition of NMDAR-EPSC amplitudes compared to stressed adults that received only air (43 ± 3% of baseline peak in AIE-Str mice versus 53 ± 2% of baseline peak in Air-Str, *t*[17] = 2.413; *p* = 0.027; Figures 8A–C). The decay time of NMDAR-EPSCs recorded in the AIE-Str group was not significantly different than that of the Air-Str group (AIE-Str weighted tau, 241 ± 27 ms versus Air-Str weighted tau, 280 ± 28 ms; *t*[17] = 1.008, *p* = 0.328), and we observed no effect of treatment on the ability of Ro 25–6981 to alter EPSC decay kinetics (% change in NMDAR-EPSC weighted tau compared to baseline: AIE-Str, -17 ± 4% versus Air-Str, -13 ± 5%; *t*[17] = 0.715, *p* = 0.485, data not shown). As these findings were reminiscent of the changes observed in adolescents immediately following chronic intermittent ethanol exposure, we next evaluated the ability of an adult stress challenge to reinstate the enhanced temporal summation observed following AIE. Again, we found a significant difference between groups (*F*[1,24] = 31.99, *p* < 0.001, Figure 8D), with AIE-Str (6 mice, *n* = 8) animals showing greater temporal summation than Air-Str animals (4 mice, *n* = 5) in response to 10 Hz (*F*[1,8] = 13.26, *p* = 0.007), 20 Hz (*F*[1,8] = 16.98, *p* = 0.003), and 40 Hz (*F*[1,8] = 5.54, *p* = 0.046) stimulation bursts (Figure 8E). Further *post hoc* comparisons revealed greater temporal summation in Air-Str animals relative to non-stressed air-controls (*F*[1,39] = 199.61; *p* < 0.001), but no difference in Ro inhibition (*t*[14] = 0.5686; *p* = 0.579). Finally, and consistent with our immediate AIE withdrawal experiments, we found that acute restraint stress had no significant influence on the LTP observed in adult males in the AIE-Str group (*n* = 11) relative to those in the Air-Str group (*n* = 7) (0–10 min post-tetanus: [*t*(16) = 0.378, *p* = 0.711], 51–60 min post-tetanus: [*t*(16) = 0.157, *p* = 0.877], data not shown). Collectively, these data suggest that stress and AIE history interact to enhance sEPSC frequency (increased glutamate release), Ro inhibition (greater GluN2Bs), and temporal summation.

The sex-specific nature of these findings was confirmed when the same stress challenge given to adult female mice yielded no difference in sEPSC frequency (Air-Str: 10 mice, *n* = 19; AIE-Str: 13 mice, *n* = 21; *t*[38] = 0.51, *p* = 0.613, Figure 7D), unlike the increase seen above in adult males with an AIE history. Instead, stress in adult female mice with a history of AIE significantly enhanced in sEPSC amplitude (*t*[38] = 3.33, *p* = 0.002, Figure 7E), indicating greater postsynaptic glutamate transmission, likely through an upregulation in AMPARs. Stress was also unable to produce a difference in the summation index of air-control mice compared to adult female mice with an AIE history (*F*[1,18] = 0.022, *p* = 0.884, Figure 8F). While the response to acute stress may be influenced by the estrous cycle (Toufexis et al., 2014), low variability in our dataset suggests this is not the reason for the lack of effect in the adult female group. Overall, these findings demonstrate that an acute stressor, but not an alcohol re-exposure, can specifically engage metaplasticity within the dlBNST of adult males with a history of AIE but not females.

## Discussion

Data from both rodent and human studies agree that alcohol consumption during adolescence correlates with a higher probability of alcohol addiction during adulthood (Grant and Dawson, 1997; Rodd-Henricks et al., 2002). This suggests that persistent neuronal adaptations occur following adolescent alcohol exposure, and that these changes predispose certain individuals to substance abuse disorders later in life. The findings of the current work support this, as we observed that AIE-induced enhancements in BNST glutamatergic tone, GluN2B signaling, and NMDAR metaplasticity could be restored following a stress challenge in adulthood. We also found that the ethanol-mediated alterations in NMDAR metaplasticity (both in adolescents and following adult stress) were specific to male mice, indicating that AIE may engage different mechanisms in the sexually dimorphic BNST.

In contrast to a previous study conducted on the adult dlBNST which report augmented LTP in animals exposed to CIE relative to air-controls (Wills et al., 2012), we observed no effect of AIE on LTP induction. The air-treated controls included in the previous study, however, displayed blunted LTP relative to treatment-naïve mice—a phenomenon suspected to be a potential consequence of chronic mild stress produced by repeated pyrazole injections and vapor chamber sessions (Wills et al., 2012). While it is difficult to make meaningful comparisons across studies, it is noteworthy that the amount of LTP observed in AIE- and air-treated mice of the current study appears similar to the LTP described in naïve adult mice of the previous report (Figure 3C of Wills et al., 2012). Numerous studies have postulated that chronic homotypic stressors given at predictable intervals may lead to habituation (Grissom and Bhatnagar, 2009). Additionally, while adolescents show an extended increase in corticosterone after acute stress, their recovery to chronic stress is more rapid compared to adults (Romeo and McEwen, 2006). The difference in findings could therefore be a result of the varying degrees by which adolescents and adults respond to stress, and not an effect of alcohol *per se*.

### AIE-Enhanced Glutamatergic Tone

Our investigation of the effects of AIE on basal glutamate transmission revealed that mice exposed to ethanol display an increase in the frequency, but not amplitude, of sEPSCs and a trend for increased frequency in mEPSCs relative to air-controls. These findings imply greater glutamate release after AIE, and are similar to a previous study conducted in the dlBNST of adult male mice following CIE (Silberman et al., 2013). Specifically, Silberman and colleagues noted that CIE-exposed mice exhibit augmented sEPSC frequency in ventral tegmentum-projecting BNST neurons and that this effect can be mimicked by exogenous application of corticotrophin releasing factor (CRF). Importantly, a CRFR1 antagonist given during ethanol withdrawal blocks the CIE-induced increase in sEPSCs, indicating that glutamate release is enhanced in the BNST through locally released CRF acting on presynaptic CRF1 receptors (Silberman et al., 2013). Similar to ethanol withdrawal, adult stress has been shown to elevate CRF signaling in the BNST (Silberman and Winder, 2013) and thus likely acts as a driver of presynaptic glutamate release. In the current study, we found that stress in adults with a history of AIE produced an increase in sEPSC frequency, and not amplitude, suggesting an enhancement of glutamatergic inputs. AIE history and adult stress also boosted NMDAR metaplasticity, postsynaptic GluN2B expression, and postsynaptic GluN2B transmission. As most neurons in the BNST are GABAergic, the region’s primary source of glutamate is through projections arising from the infralimbic and prelimbic cortex, ventral hippocampus, lateral hypothalamus, and basolateral amygdala (Ch’ng et al., 2018). These cortical inputs continue to mature during adolescence and are therefore particularly vulnerable to the effects of early-onset alcohol use (Huttenlocher, 1984; Zecevic et al., 1989; Insel et al., 1990). As several of these inputs are also sensitive to stress, they may serve as a point of convergence between the effects of AIE and adult stress. Interestingly, the distribution of frequency changes produced by AIE and AIE-Str revealed that only ∼30% of neurons had significant increases in sEPSCs (Figure 7C). Thus, it seems that the enhancing effect of AIE was restricted to certain BNST inputs.

The interaction of AIE history and adult stress might reflect disrupted activation of the HPA axis, such that AIE history affects an animal’s response to future stress. Numerous studies have demonstrated that alcohol withdrawal activates the HPA axis and that chronic alcohol leads to a blunting of the HPA axis during protracted withdrawal and future stress (Richardson et al., 2008; Reviewed in Becker, 2012). While CRF is a key neural mediator in the HPA axis, CRF has also been shown to have extra-hypothalamic effects—illustrated by its involvement in stress-induced, alcohol-mediated behaviors in the absence of HPA axis activation (Le and Shaham, 2002; Breese et al., 2004). Future studies are needed to determine if the interactive effects of AIE and stress observed in the current study involve the HPA axis.

### Dynamic Regulation of NMDARs by AIE

During acute withdrawal from AIE, we observed an increase in the temporal summation of NMDAR-mediated synaptic responses in the dlBNST, suggesting the sensitization of NMDAR transmission in a key region involved in negative affect-associated alcohol withdrawal. This enhanced NMDAR-EPSC summation may reflect disrupted glutamatergic transmission at either presynaptic (altered release probability), glial (reduced glutamate reuptake), or postsynaptic (increased GluN2B or spillover activation of perisynaptic/extrasynaptic NMDARs) sites. We also found that GluN2B-selective antagonism in AIE-treated mice produced a significant decrease in the amplitude of evoked NMDA-EPSCs relative to air-controls, but no treatment effect was observed regarding the antagonist’s ability to alter decay kinetics. Along with our Western blot findings showing a significant increase in GluN1 and GluN2B, but not GluN2A, we postulate that the augmented NMDAR response was due to increased incorporation of GluN2B subunits into heterotrimeric GluN1/GluN2A/GluN2B-NMDARs complexes, as enhancing this type of receptor would likely not alter decay kinetics (Kash et al., 2008). This GluN2B-specific upregulation is reminiscent of what has been demonstrated in the BNST of adult males, where the acute inhibitory and chronic enhancing effects of ethanol on NMDARs are also GluN2B-dependent (Kash et al., 2009; Wills et al., 2012).

**FIGURE 9:**
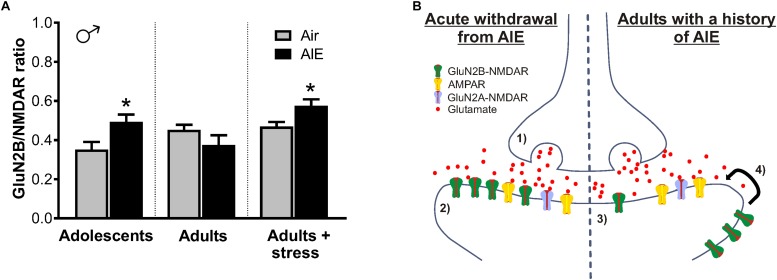
Summary of AIE-induced changes in GluN2B-containing NMDARs of the male dlBNST and a proposed model of the synaptic mechanisms underlying these changes. **(A)** GluN2B inhibition (produced by Ro 25–6981) normalized to total NMDAR transmission. Symbols indicate significant differences between groups, as determined by an unpaired *t*-test, ^∗^*p* < 0.05. Under basal conditions, adults with a history of AIE exhibited reduced GluN2B (*p* = 0.1868)—a trend that was opposite to that seen immediately following AIE and in stressed adults with a history of AIE. **(B)** Proposed model of AIE-induced changes to synaptic GluN2B-containing NMDARs during acute and protracted withdrawal, as well as during adult stress. Acute withdrawal from AIE (1) enhances presynaptic glutamate release and (2) increases in the GluN2B-containing NMDARs at the postsynaptic density. The lateral movement of these GluN2B-NMDARs to extrasynaptic locations occurs during extended withdrawal from AIE (3). Stress during extended withdrawal triggers the retargeting of extrasynaptically located GluN2B-NMDARs to the synapse (4), revealing AIE-induced changes in GluN2B-NMDAR transmission and NMDAR metaplasticity.

While the AIE-induced enhancement in GluN2B did not persist into adulthood (30 days after withdrawal), it is interesting to note that normalizing NMDAR transmission to the amount of GluN2B inhibition revealed a trend for decreased GluN2B in adult mice with a history of AIE (Figure 9A). On the other hand, restraint stress in adult males with a history of AIE produced an increase in GluN2B transmission and NMDAR plasticity, recapitulating the changes observed immediately following AIE. One potential mechanism for this is the bidirectional trafficking of GluN2B-containing NMDARs from synaptic to extrasynaptic sites. Chronic ethanol has been shown to promote the synaptic clustering of NMDARs into dendritic spines across several alcohol-sensitive brain regions (Carpenter-Hyland et al., 2004; Hendricson et al., 2007; Qiang et al., 2007) and, in hippocampal cultures, withdrawal induces the lateral diffusion of ethanol-enhanced synaptic GluN2B-NMDARs to extrasynaptic sites (Clapp et al., 2010). In line with this, a proteomic screen of GluN2B signaling in synaptic and non-synaptic fractions of the hippocampus found that withdrawal from CIE produces a dissociation of GluN2B subunits from scaffolding proteins associated with the postsynaptic density (Wills et al., 2017). Similar dynamics have been uncovered using this methodology in the hippocampus of adults with a history of AIE, where GluN2B signaling is enriched in non-synaptic versus synaptic fractions (Swartzwelder et al., 2016). While the majority of these studies have been conducted in the hippocampus, mechanisms in the adult BNST seem consistent, as changes from animals undergoing acute withdrawal from CIE appear to be, in part, due to increased transmission at extrasynaptic GluN2B-NMDARs (Wills et al., 2012).

Such NMDAR trafficking is likely a homeostatic response to changes in glutamatergic tone within the BNST. As the AIE-induced increase of sEPSC frequency is suggestive of heightened glutamate release, upregulated GluN2B-NMDARs immediately following AIE may serve to restore balance to enhanced excitatory transmission (Figure 9B, left panel). Once glutamatergic tone returns to normal, we hypothesize that GluN2Bs move to extrasynaptic sites, as they would no longer be needed, allowing them to be well-positioned to diffuse back into the synapse to respond to later stressors (Figure 9B, right panel). Since adult stress also increases glutamate through the CRF mechanism described above, such persistent changes in NMDAR plasticity during protracted abstinence—especially in a brain region critically involved in the ability of stress to modulate drug reward (Erb and Stewart, 1999; Salmaso et al., 2001)—may set the tone for later vulnerability to relapse. Considering stress during abstinence is a major contributing factor to the reinstatement of drug-seeking behavior (Becker, 2008), these findings indicate that relapse may be more difficult to prevent in adults with a history of adolescent alcohol use.

### Sex-Specific Regulation of BNST Plasticity

Another key finding of the current study was that the AIE-induced enhancement of NMDAR metaplasticity was sex-specific. The fact that there was no change in NMDAR-mediated metaplasticity in the female dlBNST may lead to the speculation that, in females, this region is not altered by early alcohol exposure. Considering the higher prevalence of anxiety and depressive disorders among women (WHO, 2017), the BNST’s role in anxiety and negative affect (Avery et al., 2016), and observations that the BNST displays greater structural connectivity in females versus males (Avery et al., 2014), this is likely not the case. Moreover, AIE-treated males and females exhibited similar elevations in sEPSC frequency during acute ethanol withdrawal and, following stress in adulthood, AIE females displayed enhanced sEPSC amplitude. These findings illustrate that a alcohol exposure in adolescent females produces long-term changes sensitive to stress, but that the mechanisms underlying these alterations are regulated via an alternate signaling pathway (e.g., by altering group 1 metabotropic glutamate receptors). While there are a number of well-documented differences in the way drugs of abuse effect males and females (Becker, 2016), the findings of the current study reveal that sexual differentiation in the neural response to addictive substances may arise during puberty, adding to the relatively limited number of studies which have described sex differences in substance use during adolescence (Kuhn, 2015). Elucidating the molecular mechanisms underlying interactions between sex, stress, and drug abuse in adolescents (as well as in adults) may aid in the development of novel and more effective therapeutic approaches to block the lasting consequences of early alcohol exposure, and may help reduce the sex-specific prevalence of certain addictive disorders.

## Data Availability Statement

The datasets generated for this study are available on request to the corresponding author.

## Ethics Statement

This study was carried out in accordance with the recommendations of the National Institutes of Health Guide for Care and Use of Laboratory Animals. The protocol was approved by the Animal Care and Use Committee at Louisiana State University Health Sciences Center.

## Author Contributions

KC and TW designed the experiments, and wrote and edited the manuscript. KC, NS, ML, MM, EH, and TW conducted the experiments.

## Conflict of Interest

The authors declare that the research was conducted in the absence of any commercial or financial relationships that could be construed as a potential conflict of interest.

## Supplementary Material

FIGURE S1 Original source data for electrophoretic blots shown in Figures 2A–F. The sample bands shown in Figures 2A–C were taken from the three blots shown in male mice section **A**, row 1. One sample was removed from data analysis because of lane contamination (indicated by the “x” in row 4 of the GluN2B column). The sample bands shown in Figures 2D–F were taken from the three blots shown in female mice section **B**, row 1.
