# Supplementary figures and images for: Regulation of NMDA Receptor Plasticity in the BNST Following Adolescent Alcohol Exposure
Source: Front Cell Neurosci. 2019 Oct 4;13:440. doi: 10.3389/fncel.2019.00440 (PMC6787153; doi:10.3389/fncel.2019.00440)

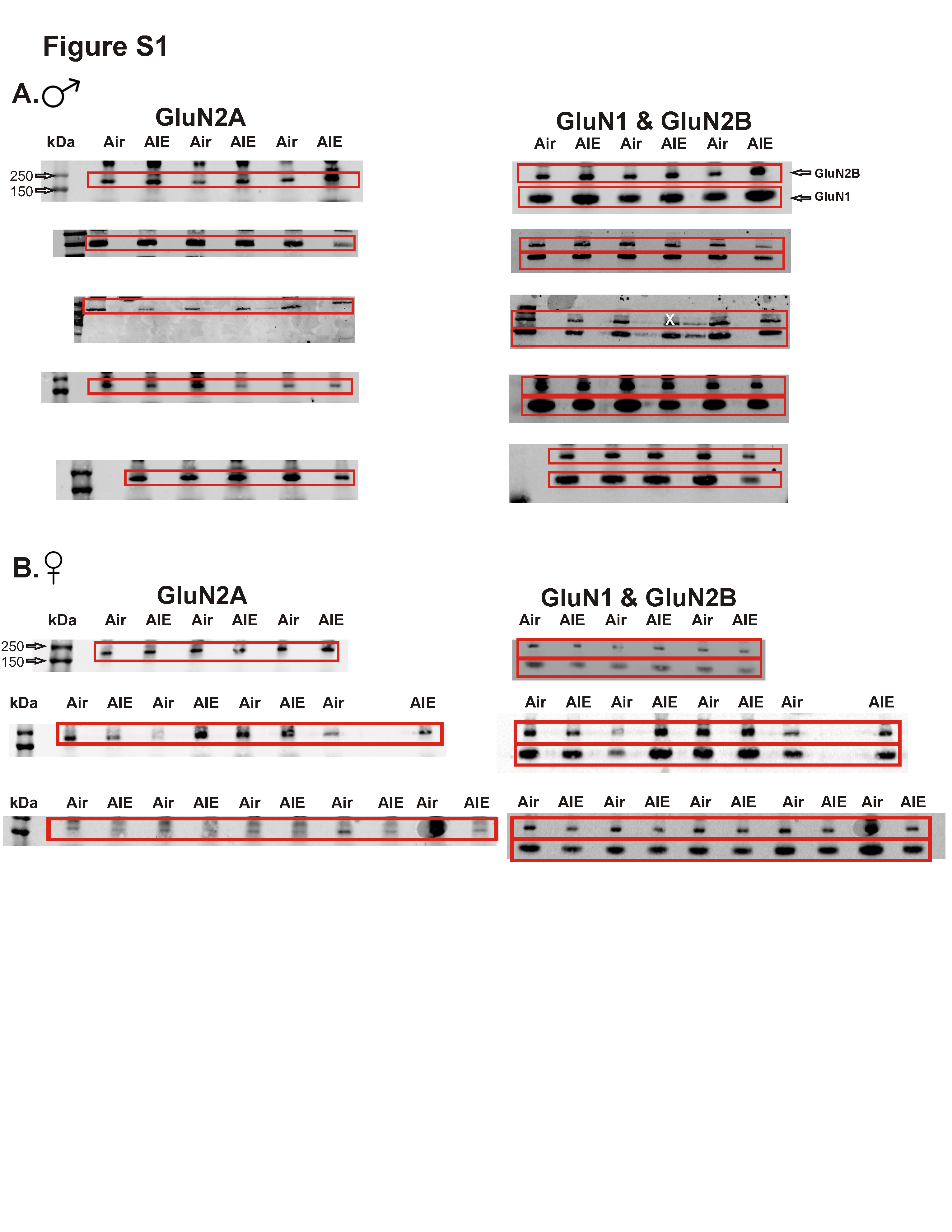

Supplement: FIGURE S1 — Original source data for electrophoretic blots shown in Figures 2A–F. The sample bands shown in Figures 2A–C were taken from the three blots shown in male mice section A, row 1. One sample was removed from data analysis because of lane contamination (indicated by the “x” in row 4 of the GluN2B column). The sample bands shown in Figures 2D–F were taken from the three blots shown in female mice section B, row 1. [file Image_1.TIFF]
